# 色谱技术在药物-血浆蛋白相互作用研究中的应用进展

**DOI:** 10.3724/SP.J.1123.2021.06028

**Published:** 2021-10-08

**Authors:** Yu BAI, Yufan FAN, Guangbo GE, Fangjun WANG

**Affiliations:** 1.中国医科大学药学院, 辽宁 沈阳 110122; 1. School of Pharmacy, China Medical University, Shenyang 110122, China; 2.中国科学院大连化学物理研究所, 中国科学院分离分析化学重点实验室, 辽宁 大连 116023; 2. CAS Key Laboratory of Separation Sciences for Analytical Chemistry, Dalian Institute of Chemical Physics, Chinese Academy of Sciences, Dalian 116023, China; 3.上海中医药大学交叉科学研究院, 上海 201203; 3. Institute of Interdisciplinary Medicine, Shanghai University of Traditional Chinese Medicine, Shanghai 201203, China

**Keywords:** 药物-血浆蛋白相互作用, 高效亲和色谱, 毛细管电泳, 综述, drug-plasma protein interactions, high performance affinity chromatography (HPAC), capillary electrophoresis (CE), review

## Abstract

小分子药物进入人体血液循环系统后与人血清白蛋白(HSA)、α_1_ -酸性糖蛋白(AGP)等血浆蛋白存在广泛的相互作用,这些相互作用深刻影响药物在体内的分布及其与靶标蛋白的结合,进而影响药物效应的发挥。深入探究药物与血浆蛋白间的相互作用对于候选药物的成药性优化、新药研发、联合用药的风险评控等意义重大。而发展高效、灵敏、准确的分析检测方法是开展药物-血浆蛋白相互作用研究的关键。近年来,色谱技术由于其高通量、高分离性能、高灵敏度等特点在该领域得到了广泛的应用,包括测定血浆蛋白翻译后修饰对药物结合的影响,多种药物的竞争性结合等。其中,高效亲和色谱(HPAC)和毛细管电泳(CE)应用最为广泛,能够通过多种分析方法获取结合常数、结合位点数、解离速率常数等相互作用信息。该文着重综述了HPAC和CE在药物-血浆蛋白相互作用研究中的常用策略及最新研究进展,包括HPAC中常用的前沿色谱法、竞争洗脱法、超快亲和提取法、峰值分析法和峰衰减分析法,以及CE中常用的亲和毛细管电泳法(ACE)和毛细管电泳前沿分析法(CE-FA)等。最后,该文还对当前色谱方法存在的不足进行了总结,并对色谱技术在药物-血浆蛋白相互作用研究领域的应用前景和发展方向进行了展望。

## Abstract (en)

After entering human blood circulation, small-molecule drugs interact extensively with various plasma proteins, such as human serum albumin and α_1_-acid glycoprotein. These interactions profoundly affect the distribution of drugs in vivo and the binding of drugs to targets, thus affecting the efficacy of drugs. In-depth investigation of drug-plasma protein interactions is of great significance for the optimization of drug properties, the development of new drugs, risk assessment, and combination therapy of drugs. Therefore, it is essential to develop highly efficient, sensitive, and accurate methods for elucidating drug-plasma protein interactions. Chromatography is a powerful tool with high throughput, high separation performance, and high sensitivity in the characterization of drug-protein interactions. High-performance affinity chromatography (HPAC) and capillary electrophoresis (CE) have been widely utilized in this field. These methods include the determination of the effects of the posttranslational modification of proteins on binding and the competitive binding of multiple drugs. In addition, various chromatographic methods are used to obtain interaction information such as the binding constant, binding-site number, and dissociation rate constant. In this review, the common strategies and recent advances in HPAC and CE in the study of drug-plasma protein interactions are briefly reviewed. The immobilization methods of proteins, the principles and applications of frontal analysis, zonal elution, ultrafast affinity extraction, peak profiling, and peak decay analysis are discussed for HPAC and affinity capillary electrophoresis (ACE) and capillary electrophoresis frontal analysis (CE-FA) for CE. HPAC relies on the fixation of proteins on the surfaces of chromatographic stationary phases by covalent linking or physical adsorption, followed by obtaining the drug-protein interaction information through a variety of chromatographic methods. In the frontal chromatography analysis, mobile phases with different concentrations of drugs are passed through the HPAC column to obtain different breakthrough times. The process can determine the number of drug binding sites and the binding constant of each site in the affinity protein with high accuracy. The zonal elution method can detect the drug binding sites on proteins using site-specific probes to determine whether there is competition between drugs and probes. The sample consumption and analysis time of the zonal elution method are much less than those in frontal chromatography analysis. The ultrafast affinity extraction method can inject complex samples, such as serum, into affinity columns to determine the free drug components. It can measure the combination and dissociation constants of drug-protein interactions by changing the chromatography flow rate. Peak profiling and peak decay analyses are both effective methods for investigating the dissociation of drugs and proteins. In CE analysis, the drug and protein samples are dissolved in an electrophoresis buffer, and their interactions are measured during electrophoresis with high accuracy and low sample consumption. However, the adsorption of proteins on the capillary wall can compromise CE performance. Common CE methods in drug-protein interaction analysis are ACE and CE-FA. ACE is usually performed by changing the effective mobility of drugs via the addition of different concentrations of proteins. This method has been widely used, and several variant techniques have been developed recently. CE-FA involves the sampling of a drug premixed at a known concentration with a target protein. Compared with other CE methods, CE-FA exhibits the unique advantages of high throughput, automatic online analysis, and the ability to determine high-order drug-protein interactions. Finally, the shortcomings of current chromatography methods are summarized, and the application prospects and development direction of chromatography technology in the field of drug-plasma protein interaction research are discussed.

大多数药物吸收入血后都会与血浆蛋白发生不同程度的可逆性结合,这种非共价相互作用主要包括静电作用、氢键作用、疏水相互作用、范德华力等^[1]^。人血清白蛋白(HSA)和α_1_ -酸性糖蛋白(AGP)都是血浆中重要的药物结合蛋白^[2,3,4]^。HSA是血浆中丰度最高的蛋白,由585个氨基酸残基组成,包含3个同源结构域(Ⅰ、Ⅱ、Ⅲ);各同源结构域又细分为两个子结构域(A和B),各子结构域分别由4和6个α-螺旋组成^[5,6]^。子结构域A和B通过脯氨酸残基提供的柔性环相对移动形成多个疏水空腔,使HSA表现出极强的药物结合能力,能与酸性、碱性和中性药物结合,进一步影响药物的转运、代谢、解毒和失活等过程^[7,8]^。HSA中已被鉴定的两个主要药物结合位点(Sudlow位点Ⅰ和Ⅱ)分别位于子结构域ⅡA和ⅢA(见图1^[9]^),具有不同的配体结合亲和力^[10,11]^。其中,位点Ⅰ空腔较大,倾向结合较大的杂环化合物和二羧酸,如华法林、吲哚美辛、水杨酸等^[12,13,14]^。位点Ⅱ空腔狭小且刚性较大,对芳香族化合物具有较高亲和力,如布洛芬、安定等^[15]^。其他HSA药物位点还包括它莫西芬位点和洋地黄毒苷位点等^[16]^。AGP占血浆总蛋白的1%~3%,能够与多种中性和碱性药物结合,具有可饱和与可置换的药物结合特性^[2]^。

**图1:**
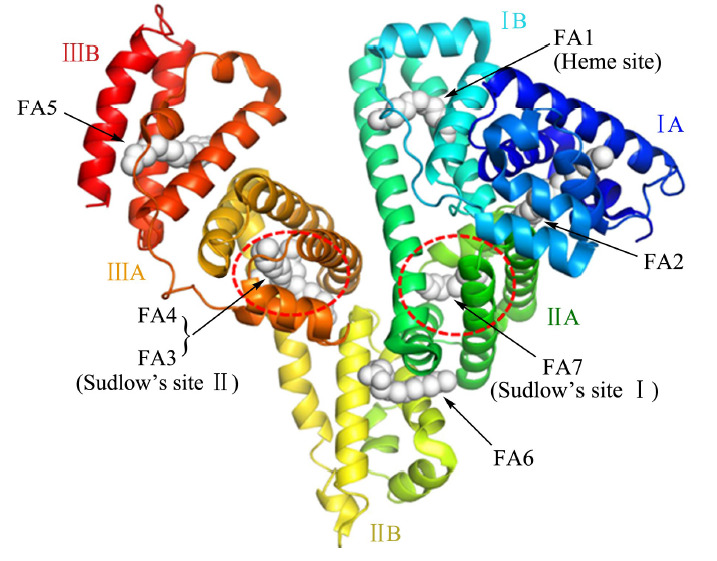
HSA结构及主要药物结合位点(以HSA-肉豆蔻酸复合物的晶体结构为例,PDB ID: 1E7G)^[9]^

药物与血浆蛋白的结合与解离通常处于动态平衡中,蛋白结合型药物作为血液中药物的一种暂时储存形式,无法实现跨膜转运。只有游离的药物分子才能通过被动扩散或各种转运蛋白跨膜运输,到达酶或受体等特定药物靶点,发挥药理活性^[17,18]^。因此,药物血浆蛋白结合率的高低,能够显著影响其药效学(PD)与药动学(PK)特性,对于药效发挥快慢、效价强度、血浆半衰期及代谢清除等过程有重要影响^[19,20,21]^。另一方面,当血浆蛋白在外周循环中与多种药物结合时,一种药物可能被另一种高浓度/强结合药物所取代,使其游离型浓度增加,药理作用或不良反应增强,最终引发药-药相互作用^[3]^。例如,磺胺类药物可在血浆蛋白结合部位竞争性置换出降血糖药甲苯磺丁脲,使后者游离型骤增,从而诱发低血糖^[22]^。药物-药物置换通常为特定结合位点的直接竞争,但也可能源于一种药物通过变构效应改变其他药物的结合^[16]^。综上所述,针对药物-血浆蛋白相互作用的研究,对于个性化精准用药、治疗药物监测及新药研发等都具有重要意义。

近年来,药物-蛋白相互作用分析检测方法得到了极大发展,主要包括:液相色谱(LC)^[23]^、超滤(UF)^[24]^、平衡透析(ED)^[25]^、质谱(MS)^[26]^、荧光光谱(FS)^[27]^、圆二色谱(CD)^[28]^、表面等离子体共振(SPR)^[29]^、核磁共振(NMR)^[30]^、等温滴定量热(ITC)^[31]^、计算机模拟^[32]^等。色谱方法具有分离效率高、应用范围广、易于自动化、能与多种检测器兼容等优点,在药物-蛋白相互作用研究中发挥着重要作用。用于表征药物-蛋白相互作用的色谱技术主要有高效亲和色谱(high performance affinity chromatography, HPAC)和毛细管电泳(capillary electrophoresis, CE)。本文主要针对这两种技术的原理、最新进展和应用进行了综述。

## 1 高效亲和色谱法

HPAC是测定弱到中等强度药物-蛋白动态相互作用的有效方法。在HPAC中,蛋白通常被固载在色谱固定相表面,进样到色谱流动相中的药物分子与固定相上的蛋白发生相互作用,通过检测药物分子保留时间、洗脱轮廓或峰面积等获取药物-蛋白相互作用信息,包括结合常数、结合位点的数量和位置、解离速率常数等^[33,34]^。

### 1.1 HPAC蛋白固定化方法

制备固载目标蛋白的色谱固定相是HPAC的首要步骤,固定相载体和蛋白固定化方法对所固载亲和蛋白的活性具有重要影响^[35]^。二氧化硅由于具有较高的机械强度、热稳定性和化学稳定性,并且粒径分布均匀,是目前广泛应用的HPAC固定相载体^[33,36,37]^。共价偶联是蛋白固载的常用策略,利用表面修饰氨基、环氧基、醛基、羟基、羧酸等功能基团的固相载体,与目标蛋白上特定氨基酸残基侧链形成共价连接^[38]^。Zhang等^[39]^将刀豆蛋白A和橙黄网胞盘菌凝集素通过还原胺化法固定在二氧化硅上,开发了一种凝集素亲和微柱,用于分离和分析AGP的糖型组分。Liang等^[40]^利用卤代烷烃脱卤酶和氯代烷烃之间的生物正交反应,将Halotag标记的血管紧张素Ⅱ1型受体共价固定到6-氯己酸衍生物修饰的氨基聚苯乙烯微球上,测定了4种药物与受体的相互作用。Li等^[41]^将β_2_-肾上腺素能受体(β_2_-AR)共价固定在*N*,*N* -羰基二咪唑活化的聚苯乙烯氨基微球上,比二氧化硅固定相具有更高的生物相容性。共价偶联方法存在多位点连接、蛋白固载取向不均一、蛋白活性降低等问题^[42,43]^。包埋法也是一种常用的蛋白固载策略,利用轻度氧化糖原作为封盖剂,通过它与肼活化载体结合将蛋白包埋于载体中,可保持蛋白质在载体中的可溶性和高活性^[43,44]^。Rodriguez等^[44]^开发了一种免疫提取/包埋系统,从血清样本中捕获HSA,通过包埋法制备亲和色谱柱,用于HSA与多种磺酰脲类药物结合的研究。

### 1.2 HPAC常用分析方法

1.2.1 前沿色谱法

前沿色谱法是一种常用的亲和色谱技术,可用于测定色谱柱容量、药物-蛋白的结合位点数量、各位点的平衡常数以及整体结合强度等^[13,45]^。前沿色谱法基本分析流程为:将药物溶解于流动相中,以固定流速通过固载目标蛋白的亲和柱,随着亲和固定相的吸附饱和,药物流出浓度逐渐增加,最终形成突破曲线(见图2);改变药物浓度,突破时间随之改变^[13,46]^。

**图2:**
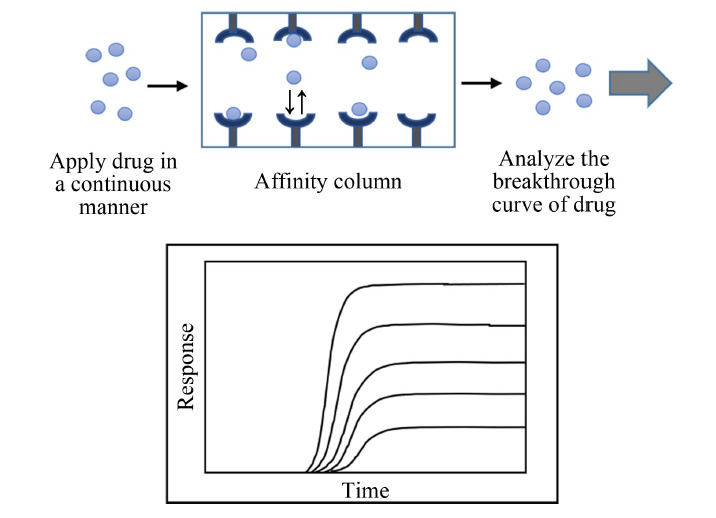
HPAC前沿色谱法进行药物-蛋白相互作用研究的示意图

当药物分子与固载蛋白为单一位点结合时,亲和柱饱和吸附量*m*_L, app_与药物浓度[A]的关系为^[47]^:


(1) $\frac{1}{m_{\mathrm{L}, \text { app }}}=\frac{1}{K_{\mathrm{a}} m_{\mathrm{L}}[\mathrm{A}]}+\frac{1}{m_{\mathrm{L}}}$

其中*K*_a_为结合平衡常数,*m*_L_为蛋白柱包含的结合位点总数。

当亲和蛋白中存在多种类型结合位点时,吸附谱图将表现出与线性响应的负偏差,需要采用非线性结合模型和方程进行数据拟合^[48]^。前沿色谱法已被应用于HSA糖化对磺酰脲类药物格列齐特、格列本脲、格列吡嗪、氯丙胺等结合影响机制的研究^[4,33,49,50]^;以及手性药物与蛋白的相互作用研究,例如普萘诺尔与低密度脂蛋白(LDL)的立体选择性结合、甲磺酸伊马替尼与牛血清白蛋白(BSA)的结合^[51,52]^等。

He等^[13]^在前沿色谱的基础上发展了多步前沿色谱法,通过采用一系列由低到高浓度的药物溶液形成多个吸附阶梯平台,测得了HSA和AGP与模型药物的结合常数。与常规前沿色谱法相比,该方法只需要在最后进行亲和色谱柱冲洗,简化了改变药物浓度所产生的多次冲洗和平衡过程,也减少了样品消耗量。此外,多步前沿色谱法与细胞膜色谱法(CMC)结合也被用于研究药物与膜受体相互作用的平衡解离常数^[53,54]^。Li等^[41]^发展了一种前沿亲和色谱-质谱(FAC-MS)与吸附能分布计算结合的方法,用于分析β_2_-AR-药物相互作用。该方法先采用FAC-MS测定沙丁胺醇、特布他林和伪麻黄碱在β_2_-AR上的原始吸附数据;再利用吸附能分布计算优化选择了3种药物与β_2_-AR结合的最佳吸附模式,分析准确度和精密度显著提高。2019年,Sun等^[55]^将前沿亲和色谱与吸附能分布计算结合,并用于研究华法林、L-色氨酸和麻黄碱在固载BSA表面的非均相吸附。

1.2.2 竞争置换洗脱法

竞争洗脱法可用于测定药物在目标蛋白上特定位点的结合^[56]^。该方法将药物溶解于流动相中,并进样一定量的特异性位点探针,如果药物和探针在固载蛋白上存在相同结合位点,探针的保留时间将随所添加药物浓度的改变而变化,由此获得药物在特定位点的相互作用信息(见图3)^[12,56]^。

**图3:**
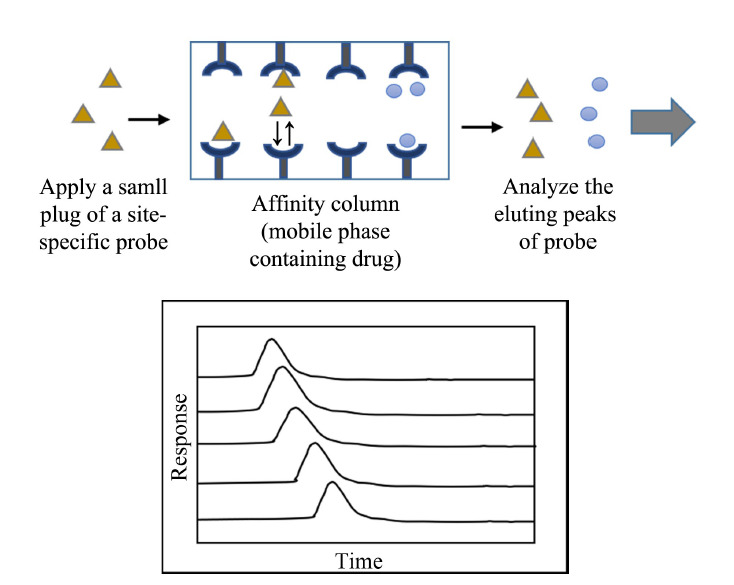
HPAC竞争洗脱法进行药物-蛋白相互作用研究的示意图

特异性位点探针的保留因子(*k*)计算公式为:


(2) $k=\frac{t_{\mathrm{R}}-t_{0}}{t_{0}}$

其中*t*_R_为探针保留时间,*t*_0_为死时间。*k*和流动相中药物浓度[I]的关系如下^[57,58]^:


(3) $\frac{1}{k}=\frac{K_{\mathrm{I}} V_{\mathrm{m}}}{K_{\mathrm{A}} m_{\mathrm{L}}}[\mathrm{I}]+\frac{V_{\mathrm{m}}}{K_{\mathrm{A}} m_{\mathrm{L}}}$

其中*m*_L_为探针与药物在固定配体上共同结合位点的物质的量,*V*_m_为柱的空隙体积,*K*_A_和*K*_I_分别是探针和药物在同一竞争位点的结合常数。当探针与药物为单位点竞争时,1/*k*与[I]为线性关系,可通过斜率和截距的比值得出药物在该位点的结合常数(即*K*_I_);如果存在多位点结合或其他相互作用,则会与预测线性关系存在偏差,这时可测得药物和蛋白的整体亲和力^[12,57]^。

竞争洗脱法已被用于检测特异性位点探针(华法林和L-色氨酸)和各种磺酰脲类药物与正常HSA和糖化HSA的结合差异^[59]^;以及研究药物和蛋白相互作用的影响因素,包括温度、pH值、离子强度、流动相的组成含量等^[56,60]^。2018年,Liu等^[61]^将β_2_-AR和电压依赖性阴离子通道蛋白1(VDAC-1)共固载于亲和色谱柱,利用竞争洗脱策略从红景天提取化合物中鉴定出红景天苷可同时结合β_2_-AR和VDAC-1,并分别确定了其结合位点和结合常数。2020年,Jeličić等^[62]^通过竞争洗脱法研究了叶酸(FA)和4种5-氨基水杨酸类药物美沙拉嗪、巴柳氮、柳氮磺胺吡啶和奥沙拉嗪对HSA相同位点的竞争结合情况。他们首先采用不同浓度FA饱和HSA特异性结合位点,随后进样一定量的5-氨基水杨酸盐,根据不同FA浓度时保留时间的变化确定FA和5-氨基水杨酸盐在HSA上存在同一结合位点的竞争^[62]^。2021年,Ovbude等^[37]^利用高性能亲和微柱和竞争洗脱法确定了降糖药瑞格列奈和那格列奈与HSA特异性位点结合情况;同时测定了HSA被乙二醛或甲基乙二醛修饰后药物在Sudlow位点Ⅰ和Ⅱ的结合变化。

前沿分析法需要向色谱柱中连续输入样品,与竞争洗脱法相比通常需要更大的样品用量以及更长的分析时间。但是前沿色谱分析中各结合位点结合常数测定之间没有相互干扰,具有更高的精确度,且能够同时确定亲和蛋白中结合位点数目和各个位点的结合常数信息^[46,47,60]^。在设计实验时,一般先采用前沿色谱分析法测定药物与蛋白质的整体结合强度,再通过特异性竞争置换洗脱确定特定位点的相互作用信息,如直接竞争、正或负变构效应、无竞争等^[33]^。

1.2.3 超快亲和提取法

超快亲和提取是一种检测药物游离组分的色谱方法,可直接在溶液体系中检测药物和蛋白的结合情况^[63,64]^。该方法将含有药物/蛋白混合物的样品上样到含有固载目标蛋白的亲和微柱上^[64]^。当流速足够高时,药物-蛋白复合物和可溶性蛋白被洗脱为非保留峰,此时可以测定原始样品中游离药物组分并计算结合常数^[65]^。当采用低至中等流速,样品流过色谱柱时,一些与可溶性蛋白结合的药物会从中解离,增加游离药物浓度,此时可测定两者的解离速率常数^[66]^。近几年,除单柱体系外,结合超快亲和萃取和双柱系统的方法也被用于测定血清等复杂样品中药物的游离组分,估算药物与蛋白的整体亲和常数^[20,67-69]^。2018年,Yang等^[70]^优化了超快亲和提取技术,分别采用单柱和双柱系统测定了几种磺酰脲类药物与正常及糖化HSA的整体亲和常数和解离速率常数,并对两种系统的优势和局限性进行了比较;结果表明双柱系统可以使用更小的游离药物组分和临床相关药物/蛋白浓度,但单柱系统操作更简单,需要的蛋白质量更少,精密度更高,且可用于估计药物-蛋白相互作用的解离速率常数。

1.2.4 峰值分析法

峰值分析法通常是以单流速或多流速注入药物,在线性洗脱条件下,测定对照柱和亲和柱上药物的保留时间和峰值方差(即谱带展宽),以此计算药物和固载蛋白的解离速率常数^[60,65,71,72]^。该方法已被用于确定HSA与L-色氨酸、卡马西平、丙咪嗪等配体的解离速率常数^[73,74]^;也被用于同时测定苯妥因的两种手性代谢物与HSA的解离情况^[71]^。2018年,Liang等^[75]^通过峰值分析法测定了5种药物沙丁胺醇、特布他林、甲氧苯胺、盐酸异丙肾上腺素和盐酸麻黄碱与β_2_-AR的解离速率常数。2020年,该方法被用于测定阿齐沙坦、坎地沙坦、缬沙坦和奥美沙坦与血管紧张素II型受体的解离速率常数^[40]^。

1.2.5 峰衰减分析法

峰衰减分析法首先采用含有药物的样品溶液使固载目标蛋白的亲和色谱柱达到饱和吸附,随后采用空白流动相进行洗脱,随时间推移检测药物的释放情况,产生一阶衰减曲线,可根据斜率测得药物的解离速率常数^[47,60,76]^。为了防止药物释放后与亲和固定相重新结合,峰衰减分析法通常使用短微柱、高流速^[77,78]^。与其他测量解离速率常数的亲和色谱方法相比,非竞争性的峰衰减分析法不需要测定塔板高度^[79]^,是高通量分析药物-蛋白解离的有效工具^[17]^。2018年,Anguizola等^[80]^通过峰衰减分析法研究了流速和洗脱pH值对几种免疫球蛋白G从亲和微柱中解离的影响。

## 2 毛细管电泳

CE作为一种高效、高灵敏的分离检测技术,可以在接近生理的条件下进行,不需要高度纯化的样品,不需要固载和标记相互作用的组分,在药物-蛋白相互作用研究中极具吸引力^[81,82,83,84]^。CE的缺点是样品中的蛋白容易吸附于毛细管壁上,稳定性较低,在分析之前需要采用NaOH、HCl或十二烷基硫酸钠(SDS)溶液清洗内壁^[85,86]^。

用于药物-蛋白相互作用研究的CE方法主要包括亲和毛细管电泳(ACE)^[87]^和毛细管电泳-前沿分析(CE-FA)^[88]^,其他方法还包括Hummel Dreyer法(HD)^[89]^、空位峰法(VP)^[90]^和空位亲和毛细管电泳法(VACE)^[91]^等。在这些方法中,CE-FA、HD、VP等方法需要采用外标或内标进行校准,而ACE和VACE法中结合参数根据药物的有效迁移率进行测定^[92]^。Michalcová等^[85]^采用ACE、CE-FA、HD法对BSA和水杨酸的相互作用进行了研究,结果表明最佳的电泳分析方法为CE-FA,它具有通量高、自动化程度高、消耗样品量少、分析时间短等优点。

### 2.1 亲和毛细管电泳法

在ACE法中,采用含有不同浓度蛋白的缓冲液作为背景电解质(BGE)充满毛细管柱,再将药物进样到毛细管内,在管内建立药物-蛋白结合动态平衡,药物的电泳迁移率在与蛋白结合后发生改变^[93,94]^。实验中常加入电渗流(EOF)标记物对药物的迁移率进行校准,抵消不同蛋白浓度下BGE黏度改变所产生的影响^[95]^。药物的有效迁移率取决于BGE中的蛋白浓度。ACE在药物-蛋白相互作用研究方面应用广泛,已被用于表征4种儿茶素与HSA和BSA的结合^[96]^,评估HSA的翻译后修饰(N-和S-同型半胱氨酸化)对HSA与儿茶素相互作用的影响并测定结合常数等^[97]^。此外,ACE也被用于手性药物与蛋白的结合研究,通常以HSA、AGP等作为固载蛋白,在实现药物对映体基线分离的同时,测定对映体与蛋白的结合常数^[1,98]^。

Li等^[99]^通过ACE法测定了10种酚类化合物与凝血酶的结合常数,进一步利用分子对接模拟确定结合位点,解析了化合物与酶活性中心的相互作用机制。Šolínová等^[100]^发展了部分填充亲和毛细管电泳法(PF-ACE),测定了人胰岛素六聚体与血清素、多巴胺、精氨酸和苯酚的结合常数。PF-ACE的优点是避免蛋白溶液随药物进入检测器,克服蛋白溶液产生的高背景信号干扰^[101]^。CE过程中蛋白结构和活性的稳定性一直是一个挑战。近年来,离子液体双水相体系(ILATPS)结合了双水相体系(ATPSs)和短链亲水性离子液体(ILs)的优点,可以增强ACE中蛋白质的稳定性,从而提高ACE方法的测量精度^[102,103,104]^。2018年,El-Hady等^[95]^采用亲水咪唑ILATPS作为运行缓冲液,提高了ACE分析过程中AGP蛋白的稳定性,精确测定了AGP与普萘洛尔、甲氨蝶呤、和长春碱的结合常数。

### 2.2 毛细管电泳前沿分析法

CE-FA具有操作简便、分析时间短,能够分析高亲和体系和多重平衡等优点^[83,105]^。CE-FA用于研究药物-蛋白相互作用时,需要游离药物和药物-蛋白复合物具有不同的电泳迁移率,且游离蛋白和药物-蛋白复合物的迁移率相近^[106]^。在CE-FA分析中,将已知浓度的药物和目标蛋白预先混合,随后进样到充满缓冲溶液的毛细管柱中,在电泳过程中将产生一个方形峰,根据峰高可获得游离药物浓度(需绘制无蛋白时的药物浓度与峰高的校准曲线)^[107,108]^。由于平台峰的高度很少受到药物迁移时间、电渗流(EOF)、毛细管长度和施加电压的影响,CE-FA方法的稳定性较好^[108]^。药物在目标蛋白上的结合位点数(*n*)和结合常数(*K*_b_)计算公式^[109,110]^为:


(4) $r=\frac{\left[\mathrm{D}_{\text {bound }}\right]}{\left[\mathrm{P}_{\text {total }}\right]}=\sum_{i=1}^{m} n_{i} \frac{K_{\mathrm{b} i}\left[\mathrm{D}_{\text {free }}\right]}{1+K_{\mathrm{b} i}\left[\mathrm{D}_{\text {free }}\right]}$

其中*r*为结合药物浓度和总蛋白浓度之比,[D_bound_]和[D_free_]分别代表结合药物浓度和游离药物浓度,[P_total_]为总蛋白浓度,*m*为蛋白上各种类型药物结合位点的总数,*n_i_*为蛋白质上等效结合位点的最大数目,*K*_bi_为相关结合常数。

CE-FA法已被用于评估正常HSA和糖化HSA与第一代磺酰脲类降糖药之间的亲和力^[83]^,以及检测常用抗凝剂华法林与HSA和BSA的高阶相互作用等研究中^[111]^。为了减少蛋白在毛细管内壁的吸附,Du等^[112]^将星形共聚物聚乙烯亚胺-接枝-聚(2-甲基-2-恶唑啉)(PEI-g-PMOXA)与聚多巴胺(PDA)共沉积于熔融石英毛细管内壁,制备了一种PEI-g-PMOXA/PDA包覆的抗蛋白吸附毛细管,通过CE-FA研究了对乙酰氨基酚与BSA的相互作用,比常规毛细管具有更好的分析准确度,与荧光光谱法结果相当。Nevídalová等^[113]^利用CE-FA法研究了双氯芬酸、布洛芬、氯丙胺和托尔布他米对HSA与L-色氨酸和利多卡因结合的影响,并测定了这些药物与HSA的结合常数。Michalcová等^[114]^基于层流廓线的横向扩散(TDLFP)和电泳介导微分析(EMMA)的原理发展了药物和蛋白混合样品在线CE-FA法,样品混合、孵化、分离的过程全部实现自动化,测定了BSA与普萘诺尔、利多卡因和酸性药物苯丁他松的结合参数,显著增加了分析通量,减少了试剂的消耗及人工操作引起的实验误差。

## 3 小结

本文所述的各种色谱方法与技术不仅可以用于药物-血浆蛋白相互作用研究,对于蛋白-蛋白、蛋白-核酸、蛋白-多糖等生物分子相互作用也具有普适性。而色谱技术在药物-血浆蛋白相互作用研究中的局限性主要包括:HPAC法需要将目标蛋白固载于色谱固定相,可能改变蛋白的构象及其药物结合性质^[115]^;在CE过程中存在蛋白在毛细管壁上的非特异性吸附、蛋白稳定性下降等情况。针对这些问题,发展更简单、高效的色谱分析方法,进一步提高药物-蛋白相互作用分析的灵敏度、准确度和通量仍然是未来研究的重点。近年来,随着各种色谱分析方法与技术的推陈出新和不断完善,以及高性能色谱-质谱联用等分析仪器的进步,为药物-血浆蛋白相互作用研究提供了更多的选择和多种技术组合,有望进一步提高分析的准确性并提供更多维度的药物-蛋白相互作用信息。
